# Immunodomination of Serotype-Specific CD4+ T-Cell Epitopes Contributed to the Biased Immune Responses Induced by a Tetravalent Measles-Vectored Dengue Vaccine

**DOI:** 10.3389/fimmu.2020.00546

**Published:** 2020-03-31

**Authors:** Tsung-Han Lin, Hsin-Wei Chen, Yu-Ju Hsiao, Jia-Ying Yan, Chen-Yi Chiang, Mei-Yu Chen, Hui-Mei Hu, Szu-Hsien Wu, Chien-Hsiung Pan

**Affiliations:** ^1^National Institute of Infectious Diseases and Vaccinology, National Health Research Institutes, Miaoli, Taiwan; ^2^Graduate Institute of Biomedical Sciences, China Medical University, Taichung, Taiwan; ^3^Graduate Institute of Medicine, Kaohsiung Medical University, Kaohsiung, Taiwan

**Keywords:** dengue, dengue vaccine, recombinant measles virus, immunodominance, envelope protein, AG129 mice

## Abstract

Dengue is an emerging mosquito-borne disease, and the use of prophylactic vaccines is still limited. We previously developed a tetravalent dengue vaccine (rMV-TDV) by a recombinant measles virus (MV) vector expressing envelope protein domain III (ED3). In this study, we used dengue-susceptible AG129 mice to evaluate the protective and/or pathogenic immune responses induced by rMV-TDV. Consistent with the previous study, rMV-TDV-immunized mice developed a significant neutralizing antibody response against all serotypes of DENV, as well as a significant IFN-γ response biased to DENV-3, compared to the vector controls. We further demonstrated that this DENV-3-specific IFN-γ response was dominated by one CD4^+^ T-cell epitope located in E_349−363_. After DENV-2 challenge, rMV-TDV-immunized mice showed a significantly lower viremia and no inflammatory cytokine increase compared to the vector controls, which had an ~100 times higher viremia and a significant increase in IFN-γ and TNF-α. As a correlate of protection, a robust memory IFN-γ response specific to DENV-2 was boosted in rMV-TDV-immunized mice after challenge. This result suggested that pre-existing DENV-3-dominated T-cell responses did not cross-react, but a DENV-2-specific IFN-γ response, which was undetectable during immunization, was recalled. Interestingly, this recalled T-cell response recognized the epitope in the same position as the E_349−363_ but in the DENV-2 serotype. This result suggested that immunodomination occurred in the CD4^+^ T-cell epitopes between dengue serotypes after rMV-TDV vaccination and resulted in a DENV-3-dominated CD4^+^ T-cell response. Although the significant increase in IgG against both DENV-2 and -3 suggested that cross-reactive antibody responses were boosted, the increased neutralizing antibodies and IgG avidity still remained DENV-2 specific, consistent with the serotype-specific T cell response post challenge. Our data reveal that immunodomination caused a biased T-cell response to one of the dengue serotypes after tetravalent dengue vaccination and highlight the roles of cross-reactive T cells in dengue protection.

## Summary

Evidence from cohort studies has shown trends of T-cell epitopes shifting to conserved regions following multiple rounds of infection, but the mechanism is unclear. Because of resource limitations, CD4^+^ T cells can recognize only a small fraction of the epitopes within a complex virus, a phenomenon called immunodominance. Immunodomination is the action of immunodominant T-cells suppressing the responses to other subdominant epitopes, which can be switched to dominant epitopes if a large amount of antigen without the immunodominant T-cell epitope is provided. The subdominant epitopes play an important role in preventing virus escape through mutations in the immunodominant T-cell epitope and have been reported to be involved in protective immunity against influenza and other viruses infection. Here, we first reported that immunodomination occurred between serotype-specific CD4^+^ T-cell epitopes after immunization with a tetravalent MV-vectored dengue vaccine, and the DENV-3 epitope became an immunodominant epitope limiting the immune responses to other serotype epitopes; however, the subdominant DENV-2-specific CD4^+^ T-cell epitope switched to a dominant epitope after DENV-2 challenge. Therefore, serotype-specific T-cell epitopes suppress each other by immunodomination but not in conserved T-cell epitopes. Our results provide a mechanism to explain why conserved dengue T-cell epitopes remain in T-cell epitope repertoires after multiple rounds of heterotypic dengue infection.

## Introduction

Dengue is the most prevalent mosquito-borne viral disease in tropical and subtropical areas, and more than half of the global population is at the risk of dengue infection (1). Approximately 390 million infections and 12,500 deaths annually are caused by the four serotypes of dengue virus (DENV-1 to 4), primarily affecting Southeast Asia and Latin America (2–4). DENV infections are usually asymptomatic or cause self-limited febrile illness but occasionally develop into dengue fever or even life-threatening severe dengue, which is characterized by plasma leakage, shock, severe bleeding, and severe organ involvement based on the WHO guidelines (5). The risk factors for severe dengue are still uncertain, but it has been proposed that antibody-dependent enhancement (ADE) and/or cross-reactive T cells are associated with the disease enhancement observed during a second heterotypic DENV infection (6–8). Although DENV-specific neutralizing antibodies provide protection from viral infection, paradoxically, as the antibody titers wane the pre-existing suboptimal antibodies facilitate DENV entry into Fcγ receptor-bearing cells (9–11), such as dendritic cells, macrophages, and monocytes, which are the major innate immune cells producing inflammatory cytokines including IL-6, TNF-α, and IL-10 during infection, which contributes to the pathogenesis of severe dengue (12, 13). Similar to ADE, the over-production of inflammatory cytokines has also been reported in cross-reactive T cells that were activated by sequence-closed but varied heterotypic dengue antigens (14, 15).

Vaccination is the most efficient approach for dengue prevention, and several dengue vaccines have been either licensed or are in clinical trials (16–21). The ideal dengue vaccine is able to elicit balanced long-term protective immunity against the four serotypes of DENV, but there are still some challenges for dengue vaccine development. One is that an imbalanced immune response or protective efficacy of live attenuated dengue vaccines has been reported in clinical trials (22, 23). A chimeric dengue vaccine (CYD-TDV) has been licensed in some countries and has shown relatively weak efficacy against DENV-2 infection (24, 25). Different replication efficiencies between the four serotypes of dengue vaccine and/or competition linked to immunodominance of T- or B-cell epitopes have been proposed to explain the imbalanced immunity (26). Immunodominance is a normal phenomenon in which, as the immune system engages with a complex antigen, such as a virus, only a small fraction of epitopes can be recognized by CD4^+^ T cells due to a limitation of resources, including antigen presenting cells, MHC class II, and space for expansion (27, 28). Another similar term is immunodomination, in which immunodominant T cells suppress the responses to other subdominant epitopes. Immunodomination has been reported in many viral infections, including influenza, vaccinia virus, and herpes virus, and is associated with protective immunity (29–32). In addition, the risk of severe dengue increased in the seronegative CYD-TDV recipients aged <9 years (33), possibly due to the waning antibodies induced by the dengue vaccine (34). This highlights the possibility of enhanced disease caused by waning antibodies in the population who have not had DENV infection before receiving the dengue vaccine and the importance of long-term safety evaluation for dengue vaccines in clinical trials.

Regardless the potential ADE, antibodies are still believed to play an important role in the protection against DENV infection, particularly since neutralizing antibody titers have been used to evaluate vaccine protection for a long time (35). In addition to the ability of antibodies to neutralize viruses, antibody avidity (36) and subclass (37) have also been reported to be associated with the severity of dengue disease. Recently, there has been increasing evidence from human and animal studies indicating that IFN-γ-producing T cells contribute to the protection against dengue virus (38–41) and highlighting the importance of the T-cell responses induced by dengue vaccination. Therefore, a long-lasting antibody response and a strong IFN-γ-dependent T-cell response are necessary for future dengue vaccines.

The currently used measles vaccine is a live attenuated measles virus (MV) with the ability to elicit long-lasting immunity in infants with few severe adverse effects (42) and has become an efficient viral vector for vaccine delivery, including flavivirus (43–45) and chikungunya (46) vaccines. We developed a tetravalent dengue vaccine (rMV-TDV) consisting of two recombinant MV vectors carrying the genes encoding bivalent fusion envelope protein domain III (ED3) of DENV-1 and -3 or DENV-2 and -4, because bivalent construct showed better immunogenicity than monovalent and tetravalent construct in ED3-based DNA vaccine. After immunization in an immunocompetent MV-susceptible YAC-CD46 mouse model, we found that rMV-TDV was able to induce both neutralizing antibodies and IFN-γ responses to the four serotypes of DENV (47); however, protection could not be evaluated because this mouse model is non-permissive to DENV replication. In this study, we used AG129 mice, which have been commonly used for studies of dengue vaccines and severe dengue pathogenesis (13, 48), to evaluate the potency of the tetravalent rMV-TDV vaccine. Our data provide comprehensive information for the protective and/or pathogenic immune responses induced by the tetravalent ED3-based dengue vaccine.

## Results

### Susceptibility of AG-hCD46 Mice to Recombinant MV Vector

**Figure 1:**
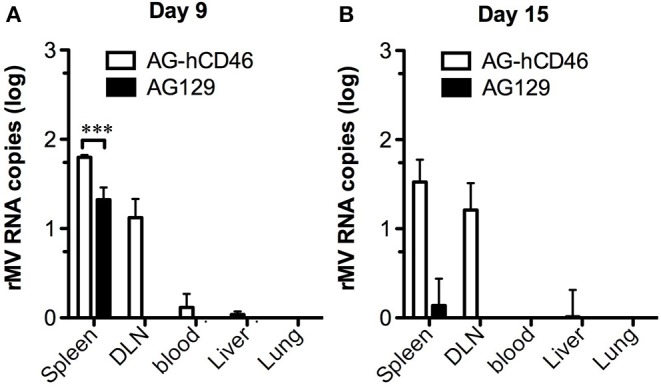
AG-hCD46 transgenic mice were more susceptible to the recombinant MV vector. AG-hCD46 transgenic and AG129 mice were intraperitoneally inoculated with 1 × 10^6^ pfu of rMV-EGFP, and total RNA from different tissues or peripheral blood cells was isolated at day 9 **(A)** or day 15 **(B)** to determine MV RNA by quantitative RT-PCR. The MV RNA copies were normalized to GAPDH RNA (1 × 10^6^ copies) and are presented as the mean ± SD of two mice. A 2-way ANOVA was used for statistical analysis (***p < 0.001).

Although AG129 mice have been used as animal models in dengue studies for many years, their lack of MV receptors is still a limitation for the evaluation of the MV-vectored dengue vaccine. To overcome this shortage, we crossbred YAC-CD46 mice, which carry the MV receptor-human CD46 transgene, into AG129 mice to obtain AG-hCD46 transgenic mice (B6.129-*Ifnar*^−/−^, *Ifngr*^−/−^ Tg(CD46); detailed in the Figure S1). To test the efficiency of rMV replication in AG-hCD46 mice, we infected AG-hCD46 mice with 1 × 10^6^ pfu rMV-EGFP by ip injection and measured the viral loads in peripheral blood and internal organs by quantitative RT-PCR. After infection, MV RNA was detected at day 9 primarily in lymphoid tissues, such as spleen, draining lymph node (DLN) and blood cells, and a significantly higher MV RNA level was observed in the spleen and DLN of AG-hCD46 mice than in AG129 mice without the transgene (Figure 1A). At day 15, only AG-hCD46 but not AG129 mice showed positive MV RNA in the spleen and DLN (Figure 1B). This suggests that AG-hCD46 mice are more susceptible to rMV than AG129 mice without the transgene.

### Both MV- and DENV-Specific Antibody Responses Were Induced by the rMV-TDV Vaccine

**Figure 2:**
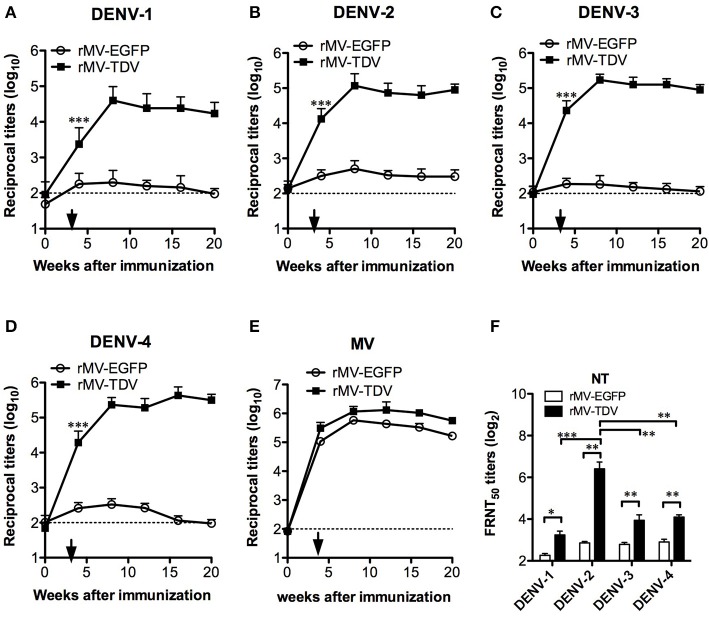
The long-lasting MV- and DENV-specific antibody responses induced by rMV-TDV immunization. Groups of AG-hCD46 mice (*n* = 5–6) were infected with 2 × 10^5^ pfu of recombinant tetravalent dengue vaccine (rMV-TDV) or vector control (rMV-EGFP) by ip injection and boosted 4 weeks later, after which sera were collected every 4 weeks until 20 weeks after immunization. **(A–D)** The specific IgG titers to DENV-1 to 4 were determined by recombinant ED3-based ELISA. **(E)** MV-specific IgG titers were measured by ELISA. **(F)** Neutralizing antibody titers to the 4 serotypes of DENV were assayed by FRNT. The results are shown as the mean ± SD, and the significance (*p < 0.05; **p < 0.01; ***p < 0.001) was analyzed by 2-way ANOVA and Student's *t*-test for the ELISA and NT assay, respectively.

To determine the immunogenicity of the tetravalent rMV-TDV vaccine, AG-hCD46 mice were immunized with either rMV-EGFP or rMV-TDV at a dose of 2 × 10^5^ pfu and boosted 4 weeks later. A significant ED3-specific IgG response was induced after a single injection of rMV-TDV, but it was not observed in rMV-EGFP-immunized mice, and the response reached peak titers after the boost and was maintained at a high level for at least 20 weeks (Figures 2A–D). There was no difference in the IgG titers between the four serotypes, except for a relatively lower IgG titer against DENV-1 compared to the other serotypes. An antibody response to the MV vector was also detected in both rMV-TDV- and rMV-EGFP-immunized mice, and no difference was observed in the IgG titers between both groups (Figure 2E). Correlated with the IgG responses, a significant increase in neutralizing antibody titers (NT) to the 4 DENV serotypes was observed in rMV-TDV-immunized mice 8 weeks after vaccination compared to the rMV-EGFP controls (Figure 2F). A significantly higher NT to DENV-2 than to the other serotypes was also observed.

### A Long-Lasting T-Cell Immune Response Was Induced by the rMV-TDV Vaccine

**Figure 3:**
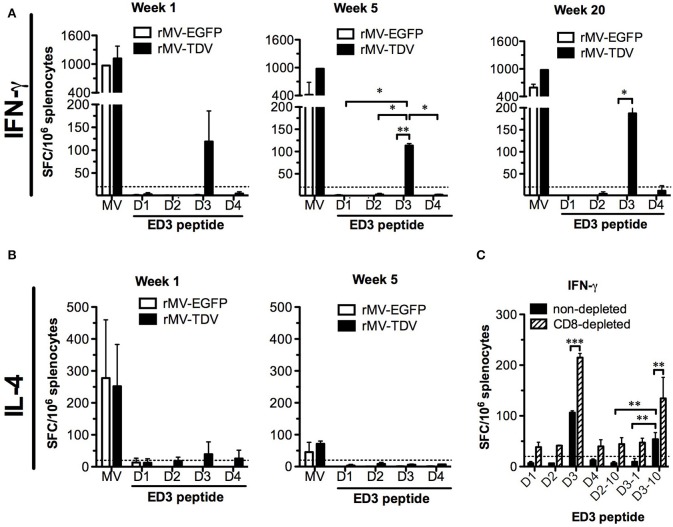
The MV- and DENV-specific memory T-cell responses induced by the MV-vectored dengue vaccine. Groups of eight AG-hCD46 mice were infected with 2 × 10^5^ pfu of rMV-TDV or rMV-EGFP by ip injection and boosted 4 weeks later. Fresh splenocytes from immunized mice (*n* = 2) were isolated at weeks 1, 5, and 20 to measure T-cell responses. MV- or DENV-1 to 4 ED3-specific IFN-γ **(A)** and IL-4 **(B)** responses after stimulation with ED3 peptide mixtures for each serotype (D1 to D4) or inactivated MV from cell lysate (MV) were assayed by ELISPOT. For the CD8-dependent T-cell response, rMV-TDV-immunized mice (*n* = 2) were sacrificed at week 6, and either CD8-depleted or non-depleted splenocytes were used for the IFN-γ ELISPOT assay **(C)**. The results represent the mean ± SD of the numbers of spot-forming cells (SFC) per million splenocytes. The Mann–Whitney *t*-test was used for statistical analyses except for the CD8 depletion assay (2-way ANOVA), and the significance (*p < 0.05; **p < 0.01; ***p < 0.001) is indicated.

To investigate the T-cell responses, we immunized AG-hCD46 mice with either rMV-EGFP or rMV-TDV and analyzed the IFN-γ and IL-4 responses at the priming (week 1), boosting (week 5), and memory (week 20) phases. Only rMV-TDV- but not rMV-EGFP-immunized mice showed an obvious increase in the ED3-specific IFN-γ response dominated by DENV-3 after a single injection (Figure 3A, left panel). Even 5 or 20 weeks later, the significantly higher level of DENV-3-specific IFN-γ responses were still maintained in rMV-TDV-immunized mice compared to the rMV-EGFP group (Figure 3A, middle and right panels). In contrast to the IFN-γ response, DENV-specific IL-4 responses were almost undetectable even after the boost (Figure 3B). Compared to the DENV responses, a 4–8 times higher levels of MV-specific IFN-γ response were induced in both the rMV-TDV and rMV-EGFP groups after priming and lasted for over 20 weeks (Figure 3A). MV-specific IL-4 responses were also induced after the prime immunization and clearly declined after the boost (Figure 3B). There was no difference in MV-specific IFN-γ or IL-4 responses between the rMV-TDV and rMV-EGFP groups. To characterize the immunogenic epitopes and CD4^+^/CD8^+^ T-cell populations responsible for the ED3-specific IFN-γ response, we used previously identified T-cell epitopes (47) to stimulate the rMV-TDV-immunized splenocytes with or without CD8^+^ T-cell depletion. A single peptide, D3-10 (DENV-3 E_349−363_; GRLITANPVVTKKEE), was able to induce a significantly higher response than others and accounted for at least half of the DENV-3-specific IFN-γ response (Figure 3C). The undetectable IFN-γ production upon stimulation with the corresponding D2-10 peptide sequence (DENV-2 E_349−363_; GRLITVNPIVTEKDS) or other serotype ED3 mixed peptides suggested that the D3-10-specific IFN-γ response is serotype-specific. After depletion of CD8^+^ T cells, the IFN-γ responses to either DENV-3 ED3 mixed peptides or D3-10 were not reduced and were even higher than those generated without CD8^+^ T-cell depletion (Figure 3C). This suggests that the DENV-3-specific IFN-γ responses are mainly independent of CD8^+^ T cells.

### Immunity Induced by rMV-TDV Vaccination Can Prevent the Disease but Not Infection by DENV-2 in AG-hCD46 Mice

**Figure 4:**
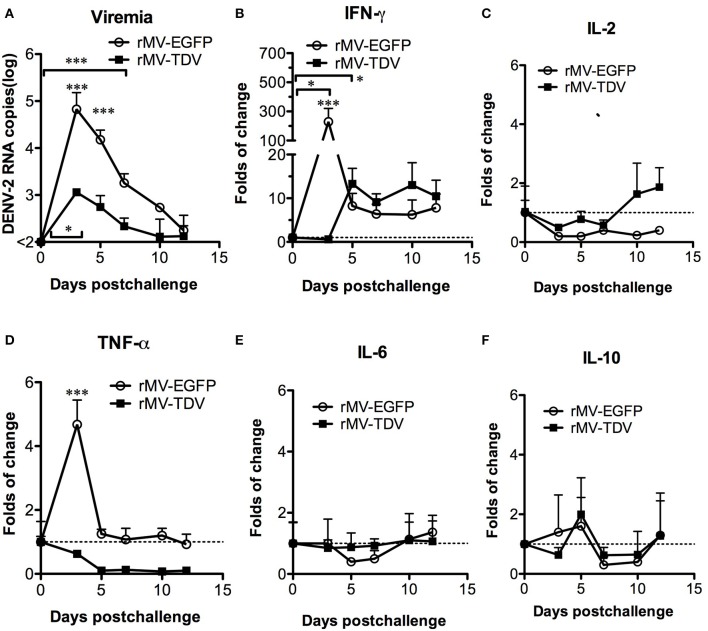
The immunization of rMV-TDV protected mice from DENV-2 challenge. Groups of AG-hCD46 transgenic mice (*n* = 4–6) were immunized with 2 × 10^5^ pfu of rMV-EGFP or rMV-TDV by ip injection and boosted 4 weeks later as indicated at the top of the figure. Eight weeks after immunization, immunized AG-hCD46 mice were challenged with 1.5 × 10^7^ ffu of DENV-2 (strain 16681) by ip injection. The viral loads **(A)** or cytokine **(B–F)** gene expression in peripheral blood cells were measured by quantitative RT-PCR with normalization to GAPDH expression. The results are shown as the mean ± SD, and the significance (*p < 0.05; ***p < 0.001) was analyzed by 2-way ANOVA, except for viremia duration, which was analyzed by 1-way ANOVA, compared to day 0.

To investigate the influence of the dominant DENV-3-specific T-cell response in the protection against heterotypic DENV, rMV-EGFP- and rMV-TDV-immunized AG-hCD46 mice were challenged with DENV-2 eight weeks later. Both rMV-EGFP- and rMV-TDV-immunized mice showed viremia peaking at day 3 and gradually declining to basal levels around day 12; however, significantly lower and shorter viremia was observed in rMV-TDV-immunized mice than in rMV-EGFP controls (*p* < 0.001; 4.82 ± 0.7 vs. 3.0 ± 0.2 log DENV-2 RNA copies at day 3 and 7 vs. 3 days of viremia duration for rMV-EGFP and rMV-TDV, respectively; Figure 4A). To understand the protective immunity and/or disease severity after DENV-2 challenge, we assayed the production of the Th1 cytokines IFN-γ and IL-2 and the inflammatory cytokines associated with severe dengue TNF-α, IL-6, and IL-10 (13) in peripheral blood cells by quantitative RT-PCR. The increase in both IFN-γ (*p* < 0.05, day 5 vs. day 0) and IL-2 in rMV-TDV-immunized mice suggested that specific Th1 responses were activated after challenge (Figures 4B,C). Interestingly, a significant extreme increase in IFN-γ (*p* < 0.001, vs. rMV-TDV) without change in IL-2 was detected in rMV-EGFP-immunized mice 3 days after challenge, but it quickly decreased below the level in rMV-TDV-immunized mice. Consistent with the higher viremia and IFN-γ increase, TNF-α but not IL-6 or IL-10 was significantly increased in the peripheral blood cells of the rMV-EGFP controls at 3 days post challenge compared to those in rMV-TDV-immunized mice, which had no increase in inflammatory cytokines (*p* < 0.001; Figures 4D–F).

### Subdominant DENV-2-Specific but Not Pre-existing DENV-3-Dominated T-Cell Responses Were Recalled After DENV-2 Challenge

**Figure 5:**
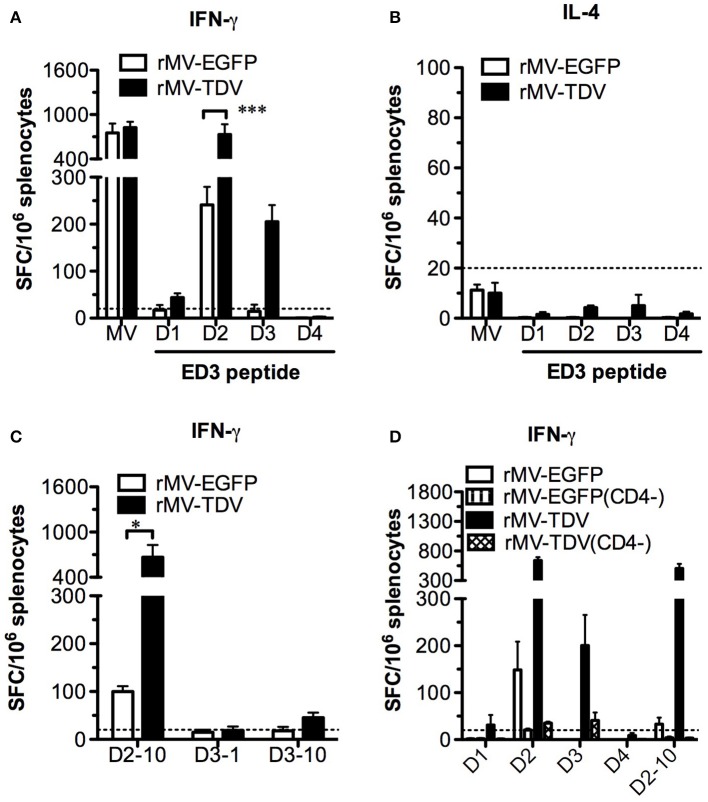
The subdominant DENV-2-specific T-cell responses were recalled after challenge. All immunized AG-hCD46 mice were sacrificed 12 days after DENV-2 challenge for the T-cell responses determined by ELISPOT. IFN-γ **(A)** or IL-4 **(B)** responses and IFN-γ responses specific to MV- or DENV-1 to −4 ED3 mixed peptides, IFN-γ responses specific to individual peptides containing T-cell epitopes **(C)** or DENV specific IFN-γ responses with (CD4^−^) or without CD4 T-cell depletion **(D)** were detected, and the results are presented as the mean ± SD of SFC per million spleen cells. The significance (*p < 0.05; ***p < 0.001) is shown.

Considering that only the DENV-3 specific T-cell response was detected after rMV-TDV vaccination together with the significant IFN-γ increase post challenge, we attempted to address whether pre-existing DENV-3-dominated T-cell responses cross-react with the heterotypic DENV-2. Surprisingly, no change in the DENV-3-specific IFN-γ responses pre and post challenge was observed in the rMV-TDV group, suggesting that the pre-existing DENV-3 ED3-specific T cells did not cross-react with DENV-2 (Figures 3A, 5A). In contrast, the robust postchallenge increase in IFN-γ responded to DENV-2 ED3 peptides (Figure 5A). Additionally, the significantly higher DENV-2-specific IFN-γ response in rMV-TDV-immunized mice than in rMV-EGFP controls (*p* < 0.05) suggested that a secondary but not primary response was induced. The results indicate that subdominant DENV-2-specific T cells were induced by the tetravalent rMV-TDV but were somehow suppressed to undetectable levels during the process of immune activation. We also measured IL-4 cytokine levels and did not find detectable ED3-specific IL-4 production in any group, similar to the prechallenge responses (Figure 5B). To better dissect the DENV-2-specific IFN-γ response, we used immunogenic peptides for stimulation. Only the D2-10 peptide showed a significantly higher IFN-γ response in rMV-TDV-immunized mice than in rMV-EGFP controls, and the number of D2-10-specific IFN-γ -producing cells was almost equal to those stimulated with DENV-2 ED3 mixed peptides (Figures 5A,C). This result suggested that the boosted DENV-2-specific IFN-γ responses were mainly specific to D2-10. Since both CD4^+^ and CD8^+^ T cells can produce IFN-γ, to rule out the possibility of D2-10 containing both CD4^+^ and CD8^+^ T-cell epitopes, we used a panel of peptides to stimulate spleen cells in the presence or absence of CD4^+^ T cells. The results demonstrated that almost all specific IFN-γ responses were diminished as CD4^+^ T cells were depleted, including responses to ED3 mixed peptides and the D2-10 peptide (Figure 5D). This result suggested that the specific IFN-γ response to D2-10 or DENV-2 ED3 mixed peptides came from CD4^+^ T cells, similar to the pattern observed in D3-10-specific CD4^+^ T cells pre challenge.

### The rMV-TDV-Induced Antibody Responses Were Reshaped by DENV-2 Challenge

**Figure 6:**
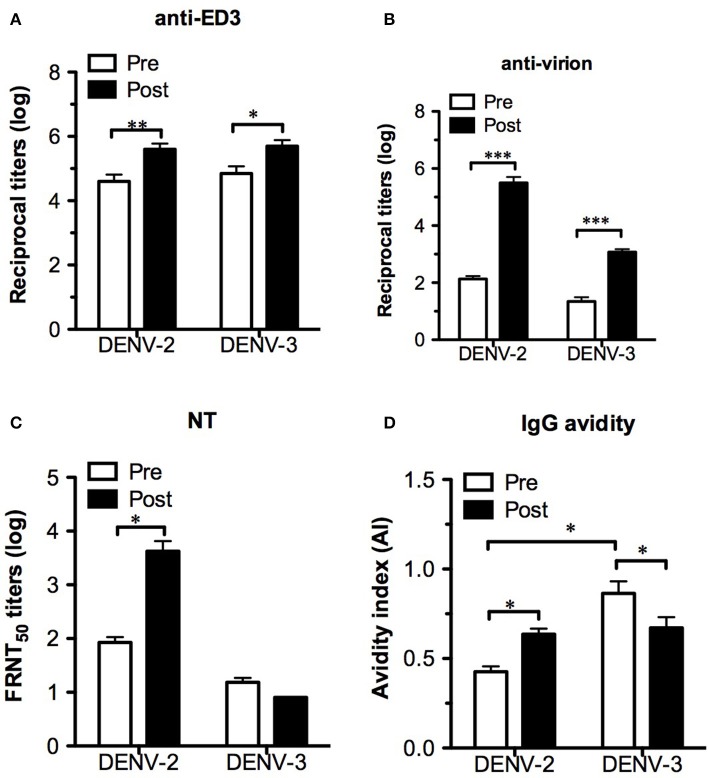
Changes in antibody responses after DENV-2 challenge. Serum samples from immunized AG-hCD46 mice 8 weeks after immunization (pre) or 12 days post challenge (post) were used for the assay. Either ED3- **(A)** or virion- **(B)** specific IgG titers were detected by ELISA. The neutralizing antibody titers **(C)** and ED3-specific IgG avidity **(D)** were measured by FRNT and avidity assays, respectively. The results are presented as the mean ± SD, and the significance (*p < 0.05; **p < 0.01; ***p < 0.001) was analyzed by 2-way ANOVA, except for IgG avidity, which was analyzed by the Mann–Whitney test.

Despite the changes in T-cell responses, it is necessary to investigate the effect of pre-existing DENV-3 dominant T cells on antibody responses after DENV-2 challenge, including antigenicity, neutralizing antibody levels and IgG avidity. To determine the IgG titers recognizing ED3 or virus particles, both recombinant ED3 and whole virions were used as ELISA antigens. After challenge, the comparable levels of IgG increase in both DENV-3 and -2 ED3-immunized mice suggested that the cross-reactive IgG responses were boosted (Figure 6A). Consistent with the ED3-specific IgG results, virion-specific IgG also demonstrated cross-reactivity and a significant increase in IgG titers against both DENV-3 and -2 virions (Figure 6B). In contrast to the cross-reactive pattern of IgG responses, the neutralizing antibody response was serotype-specific, and only DENV-2-specific NT increased significantly post challenge (Figure 6C). Although DENV-3 ED3-specific IgG had a significantly higher avidity than DENV-2 ED3-specific IgG prior to challenge, the opposite was observed for DENV-3- and -2-specific IgG avidity after DENV-2 challenge with a significant increase in IgG avidity in the DENV-2 group and a significant decrease in IgG avidity in the DENV-3 group (Figure 6D).

## Discussion

In this study, we used an AG129 mouse model to investigate the potency of the rMV-TDV vaccine. Although the protection of rMV-TDV against DENV-2 infection evidenced by reduced viremia and no inflammatory cytokine increase was encouraging, the immunodominant DENV-3 CD4^+^ T-cell responses induced by the tetravalent dengue vaccine attracted more attention. The goal of a balanced immune response against the 4 serotypes of DENV is important for the development of dengue vaccines, particularly live virus vaccines. Here, our study on the ED3-based rMV-TDV vaccine first reported how the immune system actively biased the specific CD4^+^ T-cell response to one of the 4 serotypes of DENV via immunodomination and the consequence of challenging with a heterotypic DENV. These results shed light on the importance of conserved/cross-reactive CD4^+^ T-cell responses for a dengue vaccine.

The CD4^+^ T-cell response is important for antibody production and affinity maturation (49) and contributes to viral clearance during dengue infection (39). Contrary to DENV-specific CD8^+^ T cell responses targeted to non-structural proteins, CD4^+^ T-cell epitopes are mainly located in the capsid, envelope and NS1 protein (50), including ED3 (51). ED3, with a length of ~100 amino acids, has been reported to contain serotype-specific neutralizing antibody epitopes (52) and has been used as the target antigen of dengue vaccine (53–58). In previous studies of ED3-based tetravalent DNA or MV vector vaccines, we identified serotype-specific and cross-reactive CD4^+^ T-cell epitopes within ED3 in BALB/c (H-2^d^) and C57BL/6 (H-2^b^) mice and observed immunodominance changes among these T-cell epitopes (47, 59). For example, in BALB/c mice, the tetravalent dengue ED3-based DNA vaccine induced one cross-reactive CD4^+^ T-cell epitope shared by DENV-1,−2, and−3 and one serotype-specific CD4^+^ T-cell epitope to DENV-4. After three immunizations, the serotype-specific epitope gradually decreased, but the cross-reactive epitope still remained. Another study of C57BL/6 mice showed that the CD4^+^ T-cell response induced by the tetravalent rMV-TDV vaccine was completely skewed toward DENV-3. The ranking of the responses to T-cell epitopes is called immunodominance hierarchy, in which immunodominant T cells may suppress the responses to other subdominant epitopes through competition for peptide processing and MHC binding stability or affinity between the TCR and MHC-peptide complex (60). Our current results of the immunodominant DENV-3 peptide D3-10-specific T cells in rMV-TDV-immunized AG129 mice (H-2^b^) have also demonstrated the ability to suppress the subdominant D2-10-specific CD4^+^ T-cell response, which has not been detected during rMV-TDV vaccination (containing both D3-10 and D2-10 epitopes) until the DENV-2 challenge (providing D2-10 epitope only). Consistent with the findings of subdominant CD8^+^ T cells in other virus infections (31), the subdominant DENV-2 peptide D2-10-specific CD4^+^ T-cell response was recalled following DENV-2 challenge and conferred protection with high levels of IFN-γ and IL-2 production, as well as a significant increase in DENV-2-specific NT and IgG avidity. The presence of subdominant T cells can increase the breadth of T-cell epitopes to prevent the emergence of viral escape mutants that express variant peptides in immunodominant T-cell epitopes (27). In this case, the heterotypic DENV-2 seems to be a mutant virus with a variant immunodominant epitope to pre-existing DENV-3 dominant immunity and raised a DENV-2-specific subdominant memory CD4^+^ T-cell response.

The interference between DENV-2- and -3-specific T-cell epitopes occurred not only in the MV vector vaccine but also in dengue virus infection. Weiskopf et al. has shown that in the case of both DENV-2/-3 and DENV-3/-2 heterologous infections, the previous infection with a different serotype impaired the development of responses directed to serotype-specific but not conserved/cross-reactive epitopes (61). Our results here provided further understandings that even the serotype-specific epitopes presented simultaneously, the impairment of heterologous serotype-specific responses still occurred. In addition, this interference caused by immunodominantion was observed between specific/non-cross-reactive epitopes and different to the so-called “original antigenic sin,” which has been proposed that previous DENV-priming T cells cross-react with the heterotypic epitopes during secondary heterologous infection (14, 15). Since T-cell epitopes are MHC-restricted, the immunodominant DENV-specific T-cell epitopes vary in different MHC, as we have reported that different outcomes among ED3-specific T-cell epitopes between BALB/c (H-2^d^) and C57BL/6 (H-2^b^) mice. It has also been reported that human HLA polymorphism affected the magnitude and breadth of DENV-specific CD4 T-cell responses and a lower risk of hospitalized diseases were associated with a higher magnitude of T-cell responses in some alleles (62). It suggests that comprehensive analysis of DENV-specific CD4 T-cell epitopes in different HLA-alleles is important to elucidate the pathogenesis of DENV infection and correlates of protection.

It is of particular importance for the evaluation of tetravalent live dengue vaccines if immunodomination affects the T-cell repertoire. There are four serotypes of DENV co-circulating within the same endemic regions and sharing 70% amino acid identities. The coexistence of serotype-specific and conserved/cross-reactive CD4^+^ T-cell epitopes after dengue infection is well known. It seems that the distribution of CD4^+^ T-cell epitopes varied between different serotypes of DENV (61) but shifted to the conserved/cross-reactive CD4^+^ T-cell epitopes following multiple rounds of DENV infection (63), as seen in dengue-specific CD8^+^ T cells (64). Given that the current tetravalent live dengue vaccines are usually formulated by 4 independent viruses or chimeras with the same backbone, it is possible that the immunodominance hierarchy in the tetravalent live dengue vaccine differs from that in natural infection, which was dominated by conserved/cross-reactive T-cell epitopes after sequential monovalent virus infection. Although recent clinical trial data showed that CD4^+^ T-cell responses induced by tetravalent live dengue vaccine largely focused on same epitopes as those detected in natural infection, but there were substantial serotype-specific epiotopes appeared in individuals received tetravalent live dengue vaccine (65). It is worth to consider the possibility that these serotype-specific immunodominant T-cell epitopes might suppress the other serotype-specific subdominant T-cell responses as seen in this study and/or be inhibited by the recalled subdominant T cells after a heterotypic DENV infection, as seen in the case of influenza virus (27). Based on the theory of immunodomination, boosting with an antigen containing subdominant T-cell epitopes via a DNA or protein/peptide vaccine can override the existing immunodominance hierarchy. Therefore, a heterologous prime-boost strategy with live virus and DNA/protein vaccines may be a good choice to balance the effect of immunodomination.

In addition to CD4^+^ T cells, CD8^+^ T cells also play an important role in the protection against dengue infection (66). In our experiments, we did not detect CD8^+^ T-cell responses against DENV ED3 either after rMV-TDV immunization or DENV-2 challenge. This could be due to the lack of CD8^+^ T-cell epitopes located within the ED3 region; however, exhaustion of CD8^+^ T-cell responses has been observed in virus-infected AG129 mice (67). Considering that our rMV-TDV is a live viral vector with the ability of persistent replication at least 15 days after immunization (Figure 1), it cannot rule out the possibility that T cell exhaustion is also involved.

In addition to the T-cell responses, the protective efficacy of the neutralizing antibody response also contributes to the defense against dengue. In contrast to DENV-3-dominated CD4^+^ T-cell responses, neutralizing antibodies against the 4 serotypes of DENV were induced by rMV-TDV immunization with the highest NT to DENV-2. Considering the lower viremia and no increase in inflammatory cytokines after the initial infection, the pre-existing high NT to DENV-2 substantially contributed to protection against DENV-2. The presence of subdominant DENV-2-specific CD4^+^ T cells is, of course, a reason for the high NT to DENV-2; however, the higher IgG titers against DENV-2 virion than against DENV-3 before challenge also suggested that DENV-2 ED3 is structurally mimicking virus particles. After challenge, the raised subdominant DENV-2-specific CD4^+^ T cells further enhanced the DENV-2-specific NT and IgG avidity. The previously observed correlation between IgG avidity and NT (68) agreed with our findings. Moreover, the decreased avidity of IgG against DENV-3 also correlated to the observations in human heterotypic infections (69), in which the primary infecting DENV-3-specific long-lived plasma cells were replaced with secondary infecting DENV-2-specific plasma cells.

Although the use of mice lacking both type-I and type-II IFN receptors definitely affects protective immunity, including T- and B-cell responses against DENV and other viruses, the advantages of AG129 mice, which are permissive for DENV replication and show dengue-like symptoms, are valuable for vaccine evaluation (70, 71). Compared to the subdominant DENV-1, -2, and -4 specific IFN-γ responses still detected in rMV-TDV-immunized immunocompetent B6 mice, the totally undetectable subdominant T cell responses in AG129 mice suggested that the suppression of the immunodominant DENV-3-specific T-cell responses was stronger in AG129 mice. One of the explanations for this is the increased replication efficiency of rMV-TDV in AG129 mice. It has been reported by us and others (72) that the replication of MV-vectored vaccines was decreased to a minimal level in B6 mice compared to immunodeficient IFNAR mice (73). Therefore, we chose AG129 mice expressing MV receptor-human CD46, which have a demonstrated better susceptibility to the MV-vectored vaccine than AG129 parental mice. In humans, type-I interferon cytokines are also believed to have an essential role in host immunity against viral pathogens (74, 75). However, the safety of the live attenuated measles vaccine in individuals with type-I and/or type-II interferon defects is not clear. It has been reported that some cases of inherited type-I interferon defects resulted in life-threatening complications after vaccination with live attenuated measles vaccine in previously healthy individuals (76). Paradoxically, there was also a report showing no adverse sequelae in humans with genetic defects in both the *IFNAR1* and *IFNGR2* genes after attenuated measles vaccination (77). No obvious signs associated with the MV-vectored vaccine were detected at least 20 weeks in our experiments. Of course, it is important to translate the findings in AG129 mice into clinical use with caution.

In conclusion, our studies not only demonstrated the protection of rMV-TDV against DENV-2 infection but also revealed that immunodomination may play a role in a biased immune response to one of the 4 serotypes of DENV and contribute to the shift of immunodominant T-cell epitopes from serotype-specific to conserved/cross-reactive. Immunodomination is of particular importance in live virus dengue vaccines. Based on the immunodomination theory, a heterologous boost to provide antigen with subdominant T-cell epitopes can override the influence of pre-existing immunodominant T cells. These data provide more understanding for the design of future dengue vaccines.

## Materials and Methods

### Ethics Statement

C57BL/6 mice obtained from the National Laboratory Animal Center (Taipei, Taiwan), AG129 mice (129sv *Ifnar*^−/−^*Ifngr*^−/−^) purchased from Marshell Bioresources (Hull, East Yorkshire, UK) and human CD46 transgenic YAC-CD46 mice [B6.FVB-TgN(CD46)2Ge] purchased from Jackson Laboratory (Bar Harbor, Maine U.S.A.) were housed in the animal facility of the National Health Research Institutes. AG-hCD46 [B6.129 *Ifnar*^−/−^*Ifngr*^−/−^ Tg(CD46)] mice were obtained by crossing female AG129 and male YAC-CD46 mice, and the offspring were backcrossed to AG129 mice for 4 successive generations as described in the Supplementary Material section. The genotype of AG-hCD46 mice was confirmed by genotyping PCR (Figure S1). The protocol was approved by the Animal Committee of the National Health Research Institutes (Protocol No: NHRI-IACUC-103001-A) and performed according to their guidelines.

### Recombinant Measles Viral Vectored Tetravalent Dengue Vaccine

The preparation of the tetravalent recombinant dengue vaccine rMV-TDV has been described in previous studies (47). In brief, rMV-TDV consists of two recombinant MVs (rMV-DV13 and rMV-DV24, in equivalent doses) containing the fused bivalent ED3 of DENV-1 and -3 or DENV-2 and -4, separated by a linker (three repeats of GGGGS). As a control, another recombinant MV encoding the EGFP gene (rMV-EGFP) was also included. These recombinant MVs showed comparable growth curves to their parental MV in Vero cells. For the production of the rMV-TDV vaccine, Vero cells infected with rMV-D13 or rMV-D24 (at an M.O.I. of 0.02) were harvested and lysed by freeze thawing. After centrifugation, the supernatants of the lysed cells were collected, titrated and stored at −80°C for future use.

### Immunization and Challenge

Groups of 6-8-week-old AG-hCD46 mice were immunized intraperitoneally with rMV-TDV, a mixture containing 1 × 10^5^ pfu of rMV-D13 and 1 × 10^5^ pfu of rMV-D24, or 2 × 10^5^ pfu of rMV-EGFP for the control. Mice were boosted with the same recombinant viruses and doses four weeks later. In challenge experiments, mice were challenged 4 weeks after the last immunization by subcutaneously injecting 1.5 × 10^7^ ffu of wild-type DENV-2/16681.

### Quantitative RT-PCR

The total RNA of tissue homogenate and blood cells was isolated using by TRIzol (78) (Invitrogen) and cleared by an RNA clearance kit (Qiagen), reversed transcribed to cDNA by Superscript III (Invitrogen) and stored at −80°C until use. The amounts of cDNA were detected by quantitative PCR. Briefly, specific genes were amplified (Roche LC480) using the following TaqMan primers and probes. GAPDH: CAATGTGTCCGTCGTGGATCT, GTCCTCAGTGTAGCCCAAGATG and CGTGCCGCCTGGAGAAACCTGCC for forward and reverse primers and probe; DENV-2: CAGGTTATGGCACTGTCACGAT, CCATCTGCAGCAACACCATCTC, and CTCTCCGAGAACAGGCCTCGACTTCAA for forward and reverse primers and probe; MV: GGGTACCATCCTAGCCCAAATT, CGAATCAGCTGCCGTGTCT, and CTCGCAAAGGCGGTTACGGCC for forward and reverse primers and probe; IL-6: CAAAGCCAGAGTCCTTCA, GTCCTTAGCCACTCCTTC, and CCTACCCCAATTTCCAATGCTCTCCT for forward and reverse primers and probe; TNF-α: CATCTTCTCAAAATTCGAGTGACAA, TGGGAGTAGACAAGGTACAACCC, and CACGTCGTAGCAAACCACCAAGTGGA for forward and reverse primers and probe; IL-10: AGCCGGGAAGACAATAA, GGAGTCGGTTAGCAGTA, and ACTTCCCAGTCGGCCAGAGC for forward and reverse primers and probe; IFN-γ: TCAAGTGGCATAGATGTGGAAGAA, TGGCTCTGCAGGATTTTCATG, and TCACCATCCTTTTGCCAGTTCCTCCAG for forward and reverse primers and probe; and IL-2: CCTGAGCAGGATGGAGAATTACA, TCCAGAACATGCCGCAGAG, and CCCAAGCAGGCCACAGAATTGAAAG for forward and reverse primers and probe. For GAPDH, DENV-2 and MV, the copy number was determined from the standard curve of 10^1^ to 10^6^ copies of DNA plasmids encoding the GAPDH, DENV-2 E and MV N genes, respectively. The data were normalized to the amount of 1 × 10^6^ GAPDH copies, and the results are expressed as number of copies of DENV-2 or MV RNA. For IFN-γ, IL-2, TNF-α, IL-6, and IL-10, the relative copy number was calculated by the ΔΔCt method with GAPDH as a reference. The data were normalized to 1 × 10^6^ copies of GAPDH RNA, and the fold change relative to the uninfected control (day 0) is represented.

### Enzyme-Linked Immunospot (ELISPOT) Assay

The production of IFN-γ and IL-4 by mouse spleen cells was measured using an ELISPOT assay, as described elsewhere (79). Briefly, multiscreen plates (Millipore) were coated with 2 μg/ml of anti-mouse IFN-γ or anti-mouse IL-4 antibody (all from BD Pharmingen). After the plates were washed and blocked with culture medium, 1 × 10^5^ to 5 × 10^5^ fresh mouse splenocytes were added along with 2.5 μg/ml of ED3 peptide mixtures (a panel of sixteen 15-mer peptides with 9 overlapping amino acids; Table S1) for each serotype or 5 μg/ml concanavalin A (Sigma). After 40 h of incubation, the plates were washed and incubated with a biotinylated antibody against IFN-γ or IL-4 (2 μg/ml) for 2 h at 37°C. After the plates were washed, HRP-conjugated avidin (Research Laboratory Inc.) was added and incubated for 1 h at 37°C, and the assays were developed with AEC solution (BD Pharmingen). The reaction was stopped with tap water, and the plates were analyzed using an ImmunoSpot reader with ImmunoSpot software, version 5.0.3 (CTL, Cleveland, OH). Data are presented as the number of spot-forming cells (SFCs)/10^6^ splenocytes.

### CD4 or CD8 Cell Depletion

Mouse spleens were collected and processed into single-cell suspensions. For cell depletion, splenocytes were incubated with anti-CD4- or anti–CD8-conjugated microbeads (Miltenyi Biotec), and the CD4 or CD8 T cells were negatively selected by VarioMACS separation columns (Miltenyi Biotech). FACS analysis of the remaining CD4 or CD8 T cells revealed <5% purity (not shown).

### ELISA

ED3- or virion-specific IgG titers were determined by ELISA as previously documented (80). Briefly, purified recombinant ED3 or purified virus (DENV-1 to -4 purchased from MyBioSource.com) was coated onto 96-well plates overnight and blocked with 2% bovine serum albumin (BSA) in phosphate-buffered saline (PBS) for 2 h at RT. Sera were diluted using three-fold serial dilutions (starting at 1:100) and added to the wells. Bound IgG was detected with HRP-conjugated goat anti-mouse IgG antibody. After the addition of 3,3',5,5'-tetramethylbenzidine (TMB), the absorbance was measured with an ELISA reader at 450 nm. ELISA end-point titers were defined as the serum dilution that gave an optical density (OD) value two-fold higher than the background. The serum dilution was obtained from the titration curve by interpolation. If the OD value was less than two-fold of the background at the starting dilution, a titer of 33 was used for calculations.

### Neutralization Tests

A modified focus reduction neutralization test (FRNT) was used for dengue viruses. Sera were diluted using two-fold serial dilutions (starting at 1:8), and the sera were heat inactivated prior to testing. A monolayer of BHK-21 cells in 24-well plates was inoculated with virus (DENV-1/Hawaii, DENV-2/16681, DENV-3/H-087, and DENV-4/H241; gifts from Dr. Yi-Ling Lin) that had been premixed at 4°C overnight with sera samples to a final volume of 0.5 ml. The virus titer prior to premixing was ~20–40 focus-forming units (ffu) per well. Viral adsorption was allowed to proceed for 3 h at 37°C. An overlay medium containing 2% FBS and 0.8% methylcellulose in DMEM was added at the conclusion of adsorption. After 72 h of infection, the cells were fixed for 15 min in 3.7% formaldehyde/PBS, permeabilized with 0.1% Non-idet P40/PBS for 15 min and blocked with 3% BSA/PBS for 30 min. Infected cells were detected with a monoclonal anti-dengue antibody (2H2; American Type Culture Collection, No. HB-114) that reacts with all serotypes of dengue virus. After washing with PBS, the antibody-labeled cells were detected with an HRP-conjugated secondary antibody and visualized using TMB. The numbers of ffu were counted, and the neutralizing antibody titer FRNT_50_ was calculated by the Spearman-Kamber method as the reciprocal titer that produced a 50% reduction in ffu when compared with virus alone.

### Avidity Assay

For the antibody avidity assay, the original protocol described by McCloskey et al. was modified (81). In brief, mouse sera were added to 96-well plates at a concentration previously determined to give an absorbance of ~1.0 by ELISA and allowed to bind antigen for 2 h at room temperature. After removal of the unbound antibodies, ammonium thiocyanate at concentrations ranging from 0 to 3.5 M in PBS was added to the plates and incubated for 15 min at room temperature. After washing with PBST, the remaining bound IgG was detected by ELISA as described. Antibody avidity is represented as an avidity index corresponding to the molar concentration of ammonium thiocyanate required to demonstrate a 50% reduction in absorbance.

### Statistical Analyses

All statistical analyses were performed using 2-way ANOVA with Bonferroni post-test (GraphPad Prism), unless otherwise specified. Differences with a *p*-value of < 0.05 were considered statistically significant.

## Data Availability Statement

All datasets generated for this study are included in the article/Supplementary Material.

## Ethics Statement

The animal study was reviewed and approved by the Animal Committee of the National Health Research Institutes.

## Author Contributions

T-HL, Y-JH, J-YY, C-YC, M-YC, H-MH, and S-HW performed the experiments and interpreted the data. H-WC and C-HP coordinated the experiments, interpreted the data, and designed the study. T-HL, H-WC, and C-HP wrote the manuscript.

### Conflict of Interest

The authors declare that the research was conducted in the absence of any commercial or financial relationships that could be construed as a potential conflict of interest.
