# Supplementary material for: Immunodomination of Serotype-Specific CD4+ T-Cell Epitopes Contributed to the Biased Immune Responses Induced by a Tetravalent Measles-Vectored Dengue Vaccine
Source: Front Immunol. 2020 Mar 31;11:546. doi: 10.3389/fimmu.2020.00546 (PMC7145397; doi:10.3389/fimmu.2020.00546)
Supplement: Supplementary file 1 [file Table_1.doc]

**Table S1.** ED3 (E_295-397_) and conserved NS3 (NS3_190-550_) peptides used for T cell stimulation.

| DENV-1 consensus ED3 | |  | | DENV-2 consensus ED3 | | |  | |
| --- | --- | --- | --- | --- | --- | --- | --- | --- |
| Name | A.A. sequence | |  | Name | | A.A. sequence | |  |
| D1-1 | KGMSYVMCTGSFKLE | |  | D2-1 | | KGMSYSMCTGKFKVV | |  |
| D1-2 | MCTGSFKLEKEVAET | |  | D2-2 | | MCTGKFKVVKEIAET | |  |
| D1-3 | KLEKEVAETQHGTVL | |  | D2-3 | | KVVKEIAETQHGTIV | |  |
| D1-4 | AETQHGTVLVQVKYE | |  | D2-4 | | AETQHGTIVIRVQYE | |  |
| D1-5 | TVLVQVKYEGTDAPC | |  | D2-5 | | TIVIRVQYEGDGSPC | |  |
| D1-6 | KYEGTDAPCKIPFSS | |  | D2-6 | | QYEGDGSPCKIPFEI | |  |
| D1-7 | APCKIPFSSQDEKGV | |  | D2-7 | | SPCKIPFEIMDLEKR | |  |
| D1-8 | FSSQDEKGVTQNGRL | |  | D2-8 | | FEIMDLEKRHVLGRL | |  |
| D1-9 | KGVTQNGRLITANPI | |  | D2-9 | | EKRHVLGRLITVNPI | |  |
| D1-10 | GRLITANPIVTDKEK | |  | D2-10 | | GRLITVNPIVTEKDS | |  |
| D1-11 | NPIVTDKEKPVNIEA | |  | D2-11 | | NPIVTEKDSPVNIEA | |  |
| D1-12 | KEKPVNIEAEPPFGE | |  | D2-12 | | KDSPVNIEAEPPFGD | |  |
| D1-13 | IEAEPPFGESYIVVG | |  | D2-13 | | IEAEPPFGDSYIIIG | |  |
| D1-14 | FGESYIVVGAGEKAL | |  | D2-14 | | FGDSYIIIGVEPGQL | |  |
| D1-15 | VVGAGEKALKLSWFK | |  | D2-15 | | IIGVEPGQLKLNWFK | |  |
| D1-16 | KALKLSWFKKGSS | |  | D2-16 | | GQLKLNWFKKGSS | |  |
|  |  | |  |  |  | |  | |
| DENV-3 consensus ED3 | | |  | DENV-4 consensus ED3 | | |  | |
| Name | A.A. sequence | |  | Name | | A.A. sequence | |  |
| D3-1 | KGMSYAMCLNTFVLK | |  | D4-1 | | KGMSYTMCSGKFSID | |  |
| D3-2 | MCLNTFVLKKEVSET | |  | D4-2 | | MCSGKFSIDKEMAET | |  |
| D3-3 | VLKKEVSETQHGTIL | |  | D4-3 | | SIDKEMAETQHGTTV | |  |
| D3-4 | SETQHGTILIKVEYK | |  | D4-4 | | AETQHGTTVVKVKYE | |  |
| D3-5 | TILIKVEYKGEDAPC | |  | D4-5 | | TTVVKVKYEGAGAPC | |  |
| D3-6 | EYKGEDAPCKIPFST | |  | D4-6 | | KYEGAGAPCKVPIEI | |  |
| D3-7 | APCKIPFSTEDGQGK | |  | D4-7 | | APCKVPIEIRDVNKE | |  |
| D3-8 | FSTEDGQGKAHNGRL | |  | D4-8 | | IEIRDVNKEKVVGRI | |  |
| D3-9 | QGKAHNGRLITANPV | |  | D4-9 | | NKEKVVGRIISSTPF | |  |
| D3-10 | GRLITANPVVTKKEE | |  | D4-10 | | GRIISSTPFAENTNS | |  |
| D3-11 | NPVVTKKEEPVNIEA | |  | D4-11 | | TPFAENTNSVTNIEL | |  |
| D3-12 | KEEPVNIEAEPPFGE | |  | D4-12 | | TNSVTNIELEPPFGD | |  |
| D3-13 | IEAEPPFGESNIVIG | |  | D4-13 | | IELEPPFGDSYIVIG | |  |
| D3-14 | FGESNIVIGIGDKAL | |  | D4-14 | | FGDSYIVIGVGDSAL | |  |
| D3-15 | VIGIGDKALKINWYK | |  | D4-15 | | VIGVGDSALTLHWFR | |  |
| D3-16 | KALKINWYKKGSS | |  | D4-16 | | SALTLHWFRKGSS | |  |
