# Supplementary material for: Immunodomination of Serotype-Specific CD4+ T-Cell Epitopes Contributed to the Biased Immune Responses Induced by a Tetravalent Measles-Vectored Dengue Vaccine
Source: Front Immunol. 2020 Mar 31;11:546. doi: 10.3389/fimmu.2020.00546 (PMC7145397; doi:10.3389/fimmu.2020.00546)
Supplement: Supplementary file 2 [file Image_1.pdf]

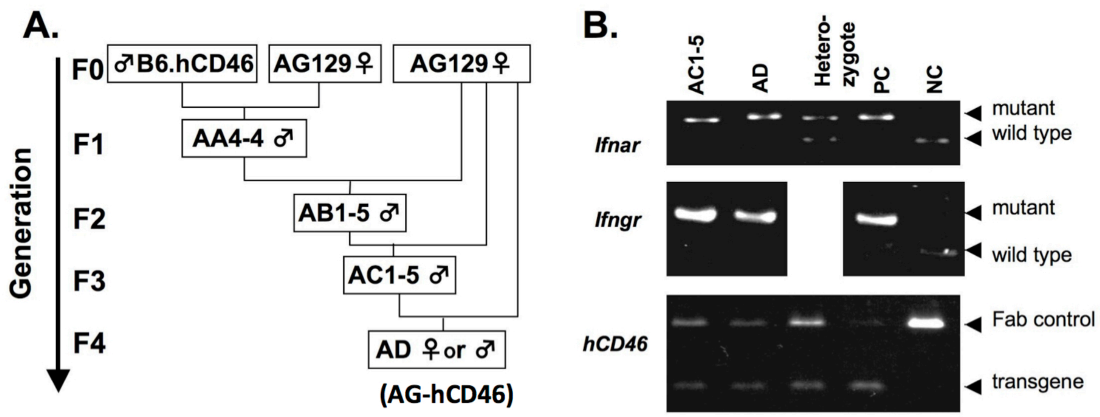

Fig. S1 Generation of AG129-hCD46 mice for MV-vectored dengue vaccine evaluation.

(A) Schematic diagram of breeding history for AG-hCD46 (B6.129 *Ifnar*$^{-/-}$ *Ifngr*$^{-/-}$ TgN(CD46)) mice is shown. The male YAC-CD46 mice (B6.hCD46) were bred to female AG129 mice, and the 1st generation of male offspring (AA4-4) with the CD46 transgene was backcrossed to AG129 mice again. The 2nd generation of male offspring (AB1-5), which carried the heterozygous *Ifnar* mutant allele and CD46 transgene by genotyping, was backcross to AG129 mice to obtain the 3rd generation (AC1-5) homozygous for both the *Ifnar* and *Ifngr* mutant alleles and the CD46 transgene. The 4th generation offspring backcrossed with AC1-5 and AG129 mice were named AG-hCD46 and used for subsequent experiments.

(B) The chromosome DNA from two AC1-5 and AD, *Ifnar* heterozygote, positive (PC) and negative control (NC) mice were isolated, and the mutant and wild-type form of *Ifnar* and *Ifngr*, hCD46 transgene and housekeeping gene *Fab* control were amplified by PCR with the specific primer sets. The genotype of AD was identified as *Ifnar*$^{-/-}$ *Ifngr*$^{-/-}$ *hCD46tg*$^{+}$.
